# AIDS-Related Stigma and Mental Disorders among People Living with HIV: A Cross-Sectional Study in Cambodia

**DOI:** 10.1371/journal.pone.0121461

**Published:** 2015-03-25

**Authors:** Siyan Yi, Pheak Chhoun, Samedy Suong, Kouland Thin, Carinne Brody, Sovannary Tuot

**Affiliations:** 1 Research Department, KHANA, Phnom Penh, Cambodia; 2 Public Health Program, College of Education and Health Sciences, Touro University California, Vallejo, California, United States of America; University of Missouri-Kansas City, UNITED STATES

## Abstract

**Background:**

AIDS-related stigma and mental disorders are the most common conditions in people living with HIV (PLHIV). We therefore conducted this study to examine the association of AIDS-related stigma and discrimination with mental disorders among PLHIV in Cambodia.

**Methods:**

A two-stage cluster sampling method was used to select 1,003 adult PLHIV from six provinces. The People Living with HIV Stigma Index was used to measure stigma and discrimination, and a short version of general health questionnaire (GHQ-12) was used to measure mental disorders. Multivariate logistic regression analysis was conducted.

**Results:**

The reported experiences of discrimination in communities in the past 12 months ranged from 0.8% for reports of being denied health services to 42.3% for being aware of being gossiped about. Internal stigma was also common ranging from 2.8% for avoiding going to a local clinic and/or hospital to 59.6% for deciding not to have (more) children. The proportions of PLHIV who reported fear of stigma and discrimination ranged from 13.9% for fear of being physically assaulted to 34.5% for fear of being gossiped about. The mean score of GHQ-12 was 3.2 (SD = 2.4). After controlling for several potential confounders, higher levels of mental disorders (GHQ-12≥ 4) remained significantly associated with higher levels of experiences of stigma and discrimination in family and communities (AOR = 1.9, 95% CI = 1.4–2.6), higher levels of internal stigma (AOR = 1.7, 95% CI = 1.2–2.3), and higher levels of fear of stigma and discrimination in family and communities (AOR = 1.5, 95% CI = 1.1–2.2).

**Conclusions:**

AIDS-related stigma and discrimination among PLHIV in Cambodia are common and may have potential impacts on their mental health conditions. These findings indicate a need for community-based interventions to reduce stigma and discrimination in the general public and to help PLHIV to cope with this situation.

## Introduction

Despite care and treatment advances that have turned HIV into a chronic and manageable condition, people living with HIV (PLHIV) continue to suffer from stigma and discrimination from their family and communities. AIDS-related stigma and discrimination impede millions of PLHIV from accessing and benefiting from effective prevention and treatment services [1]. As a result, approximately 50–60% of HIV-infected people are unaware of their sero-status, and many choose to hide it [2]. Furthermore, AIDS-related stigma and discrimination have been found to be associated with delays in seeking care [3] and potential barriers to HIV counseling and testing [4], disclosure of HIV sero-status [5,6], retention in care and treatment [7], and uptake of and adherence to antiretroviral therapy (ART) [8,9]. There is also mounting evidence that AIDS-related stigma and discrimination are associated with other social outcomes such as racism, poverty, and heterosexism [10–12].

Mental disorders are also among the most common problems in the life of PLHIV regardless of gender or race/ethnicity and can impact their health status, healthcare seeking behaviors, and quality of life. Depression, alcohol use disorders, and neurocognitive disorders are the most prevalent mental problems in this vulnerable population [13]. Studies in different countries have reported point prevalence rates of major depressive disorder among PLHIV from 3% to 54% [14–21]. Using the Composite International Diagnostic Inventory, a national survey in South Africa found that 44% of PLHIV had a diagnosable mental disorder [22]. Of these, major depression accounted for 11%, mild depression for 30%, and alcohol abuse for 12%. Furthermore, compared to HIV-negative individuals, PLHIV are two to three times more likely to develop mental disorders [23,24]. Mental disorders are also associated with several health and healthcare seeking behaviors such as poor adherence to medications [25–27], low rates of retention in ART care [28], and poor ART-related clinical outcomes [29]. PLHIV suffering from depression progress faster from HIV to AIDS compared to non-depressed PLHIV [30,31].

Several biological and socioeconomic factors have been found to be associated with mental disorders in PLHIV such as depression and anxiety. These factors include compromised immune system and increased opportunistic infections [32], absence of ART [33], perceived social support [34], and death of significant other due to AIDS [35]. Similar to general population, several socio-demographic variables such as older age, female gender, low education, and unemployment have also been found to be related to mental disorders among PLHIV [36,37].

The double burden of AIDS-related stigma and mental disorders could result in a number of problems in health and quality of life for PLHIV. Previous studies have also linked AIDS-related stigma and discrimination to mental wellbeing of PLHIV. Steward and colleagues found that all forms of stigma and discrimination are ultimately associated with depressive symptoms among PLHIV in India [38]. Several more recent studies have confirmed these findings and included other mental health outcomes such as anxiety, stress, or posttraumatic stress disorders (PTSD) in different populations of PLHIV in different countries around the world including mainland China [33,34], India [39], South Africa [40], the United States [41–42], and several other countries [5].

Despite this alarming situation, AIDS-related stigma and discrimination as well as mental health issues among PLHIV in many developing countries have received only limited attention, and the linkage between these two common conditions has not been well addressed. To the best of our knowledge, no study of the relationship between AIDS-related stigma and mental health conditions has been conducted in Cambodia, where success stories have been touted in HIV prevention, care, and treatment programs with more than 95% of PLHIV in need of ART currently on the treatment [43]. We therefore conducted this study to examine the prevalence of AIDS-related stigma and discrimination and its relationship to mental disorders among PLHIV in Cambodia. We hypothesized that PLHIV who have experienced higher levels of stigma and discrimination in family and communities would express higher levels of mental disorders after controlling for the effects of potential confounding factors explored in previous studies.

## Materials and Methods

### Ethical statement

Participation in this study was voluntary, and a written informed consent was obtained from each study participant after a detailed description of study objectives and procedures was provided. Moreover, the study participants had an opportunity to refuse or to discontinue participation at any time. Privacy was strictly protected by conducting the interviews at a private place, and we ensured confidentiality of the respondents by removing all personal identifiers from the survey questionnaires. This study was approved by the National Ethics Committee for Health Research, the Ministry of Health, Cambodia (No. 082NECHR).

### Study sites and sampling

Data used in this study were collected in May 2014 as part of an impact evaluation study for the Sustainable Action against HIV and AIDS in Communities (SAHACOM) Project. Participants included 1,005 PLHIV living in six provinces including Battambang, Kampong Cham, Pailin, Pursat, Siem Reap, and Takeo. Coordination and administration of the survey were facilitated by community-based organizations with support from provincial health departments and local authorities to ensure the effectiveness and quality of the data collection.

A two-stage cluster sampling method was utilized to select the study samples. The sample size was proportionally allocated to the size of PLHIV in each province. The number of health centers in each selected province to be included in the study was decided based upon the number of PLHIV registered in each health center. In order to be included in the study, a health center must have at least 20 PLHIV registered. In addition, other factors were assessed when deciding whether to include a health center in the study such as convenience for data collection and duration of the project implementation in the health center coverage. We then used the probability proportional-to-size sampling to select the required number of PLHIV from each province. The inclusion criteria for the study participants were: (1) men or women aged 18 years or older; (2) having been registered as a PLHIV within the network of a community-based implementing partner; (3) being able to provide consent to participate in the study; and (4) being able present themselves on the day of the data collection. PLHIV were excluded if they were mentally and/or physically too sick to participate in the study.

### Data collection training and procedures

Prior to the data collection, all research team members were trained for three days on data collection methods including tool pretesting and reflection. The main objective of the training was to ensure that all interviewers and field supervisors understood the procedures and followed the standardized guidelines in the same manner to guarantee quality of the data. The training covered necessary skills including interview techniques, confidentiality, and practices of the questionnaire administration. We also reviewed the study protocol during the training sessions in order for the team members to be thoroughly familiar with it. Quality control skills such as rechecking and reviewing the questionnaires after administration as well as resolving issues that might arise during the fieldwork were included in the training. Regular review sessions with interviewers were conducted during the survey period to review progress and communicate any problems or issues occurring during the data collection.

### Questionnaire development

The questionnaire was developed using standardized and validated tools adapted from previous studies. The questionnaire was initially developed in English and then translated into Khmer, the national language of Cambodia. Another translator back-translated it into English to ensure that the “content and spirit” of every original item would be maintained. Clear instructions and explanations were addressed to avoid any confusion during the interviews.

A pilot study was conducted before constructing the final questionnaire to ensure that the wording and contents were culturally suitable, acceptable, and clearly understandable for the study participants. In the pilot study, face-to-face interviews were conducted with 10 samples randomly selected from a pre-ART/ART site in Phnom Penh to assess the contents, format, length, language, and appropriateness of the questionnaire. Necessary modifications were made based upon feedbacks from the pilot study and comments from researchers and practitioners working on HIV in Cambodia.

### Variables and measurements

Socio-economic characteristics of the participants and other variables such as social support and pre-ART/ART services received in the past 12 months were measured using existing tools adapted from our previous studies in the same population [44] and the most recent Cambodia Demographic and Health Survey [45].

Mental disorders were measured using a short version of the General Health Questionnaire (GHQ-12) developed by David Goldberg [46]. It is a screening tool used to screen mental health problems experienced by an individual in the past few weeks. GHQ-12 has also been validated in Asian populations [47,48]. Each item of the GHQ-12 were rated on a 4-point Likert scale, with the response options of “0 = less than usual,” “1 = no more than usual,” “2 = rather more than usual,” or “3 = much more than usual.” Goldberg & William suggested the use of scoring method ‘0-0-1-1’ as this particular method is believed to help eliminate biases resulted from respondents who tend to choose responses 0 and 3 or 1 and 2 [49]. The mean score for the whole population (3/4) was used as the cut-off for defining lower and higher levels of mental disorders as it provides a rough guide to the best threshold [50]. The Cronbach’s alpha for the scale among PLHIV in this study was 0.72.

Perceived AIDS-related stigma and discrimination were measured using the People Living with HIV Stigma Index that has been developed by and for PLHIV [51]. It is a result of a partnership between the International Planned Parenthood Federation, the International Community of Women Living with HIV, UNAIDS, and the Global Network of People Living with HIV. The questionnaire is divided into three main sections: (1) experiences of stigma and discrimination in different settings such as home, community, workplace, religious settings, and healthcare facilities (18 items); (2) internal stigma (16 items); and (3) fear of stigma and discrimination in family and communities (5 items). The yes/no questions asked respondents to report their experiences, perception, and feeling related to stigma and discrimination in the past 12 months. The Cronbach’s alpha for the sub-scales were 0.78, 0.75, and 0.77, respectively.

### Data analyses

Data were coded and entered into a computerized database using EpiData version 3 (Odense, Denmark). Double data entry was performed to minimize entry errors. Chi-square test was used to compare socio-demographic characteristics, experiences of AIDS-related stigma and discrimination, internal stigma, and fear of stigma and discrimination among respondents with lower (GHQ-12≤ 3) and higher (GHQ-12≥ 4) levels of mental disorders. Total scores were calculated for experiences of stigma and discrimination, internal stigma, and fear of stigma and discrimination, and then the mean score of each scale was used to divide respondents into two groups for comparisons. Bivariate logistic regression analysis was used to examine the relationship of levels of stigma and discrimination, internal stigma, and fear of stigma and discrimination with levels of GHQ-12. Multivariate logistic regression models were then constructed to examine the association between each stigma and discrimination sub-scale with levels of GHQ-12 controlling for the effects of potential confounders. All variables found to have significant association with GHQ-12 in bivariate analyses at a level of *p*< 0.10 were simultaneously included in the models. SPSS version 22 (IBM Corporation, New York, USA) was used for all data analyses.

## Results

### Socio-demographic characteristics

In total, 329 men (32.8%) and 674 women (67.2%) were included in this study, with a mean age of 42.8 years (SD = 8.8 years). Two respondents were excluded from the analyses because their responses to GHQ-12 questionnaire were not completed. The majority of the participants were married (62.8%); while 35.2% were divorced, separated, or widowed; and only 2.0% were never-married. Their main occupations included farmers (39.0%), self-employed (21.6%), laborers (17.7%), office workers (3.9%), or other (5.4%). Mean monthly income of the respondents was US$75, and the majority of them (95.2%) have received some forms of social support in the past 12 months. On average, participants in this study have been living with HIV for 8.4 years (SD = 4.5 years). Regarding HIV status disclosure, 66.0% of the respondents reported that their spouse or partner was also HIV infected, and the majority of their spouse or partner (79.0%) and family members (97.7%) knew their HIV status. For HIV treatment and care, 96.4% of respondents reported being currently on ART with a mean CD4 count at the most recent blood test of 513 cells/mm^3^ (SD = 302 cells/mm^3^).

As shown in **Table 1**, PLHIV with higher levels of mental disorders (GHQ-12≥ 4) were significantly more likely to be female (OR = 2.2, 95% CI = 1.6–2.9), to have completed <5 years of formal education (OR = 1.4, 95% CI = 1.1–1.9), to be currently unmarried (OR = 1.4, 95% CI = 1.1–1.7), to be currently unemployed (OR = 1.9, 95% CI = 1.3–2.7), to have lower income (OR = 1.5, 95% CI = 1.1–1.5), to live with an HIV-infected spouse or partner (OR = 1.6, 95% CI = 1.1–2.3), to report that their spouse or partner knew their HIV status (OR = 1.5, 95% CI = 1.1–2.5), and to have not received any social support in the past 12 months (OR = 1.7, 95% CI = 1.1–3.0).

**Table 1:** Comparisons of socio-demographic characteristics of PLHIV with lower and higher levels of mental disorders.

Socio-demographic characteristics		Total score of GHQ-12		
		≤ 3	≥ 4	
	Total	*n* (%)	*n* (%)	OR (95% CI)
Gender				
Male	329 (32.8)	242 (38.8)	85 (22.5)	Ref
Female	674 (67.2)	381 (61.2)	292 (77.5)	2.2 (1.6–2.9)
Age group				
≤ 43 years	510 (50.7)	320 (51.3)	190 (50.3)	Ref
> 43 years	495 (49.3)	304 (48.7)	188 (49.7)	1.1 (0.8–1.3)
Formal education completed				
≥ 5 years	567 (56.4)	293 (47.0)	144 (38.1)	Ref
< 5 years	438 (43.6)	331 (53.0)	234 (61.9)	1.4 (1.1–1.9)
Marital status				
Married	630 (62.8)	405 (65.1)	223 (59.0)	Ref
Unmarried	373 (37.2)	217 (34.9)	155 (41.0)	1.4 (1.1–1.7)
Current employment status				
Employed	880 (87.6)	563 (90.2)	314 (83.3)	Ref
Unemployed	124 (12.4)	61 (9.8)	63 (16.7)	1.9 (1.3–2.7)
Average monthly income				
> US$75	565 (56.8)	277 (44.8)	152 (40.6)	Ref
≤ US$75	430 (43.2)	341 (55.2)	222 (59.4)	1.5 (1.1–1.5)
Duration of living with HIV				
≤ 8 years	520 (51.7)	328 (52.6)	891 (50.5)	Ref
> 8 years	485 (48.3)	296 (47.4)	187 (49.5)	1.1 (0.8–1.4)
Spouse/partner’s HIV status (for married PLHIV)				
Negative	197 (22.9)	141 (25.9)	56 (15.9)	Ref
Positive	663 (77.1)	404 (74.1)	256 (82.1)	1.6 (1.1–2.3)
Spouse/partner knows your HIV status				
No	77 (8.8)	54 (9.9)	22 (6.9)	Ref
Yes	794 (91.2)	494 (90.1)	298 (93.1)	1.5 (1.1–2.5)
Currently on ART treatment				
No	36 (3.6)	26 (4.2)	10 (2.7)	Ref
Yes	958 (96.4)	589 (95.8)	366 (97.3)	1.6 (0.8–3.4)
CD4 count at the most recent blood test				
> 500	512 (51.8)	304 (49.4)	170 (45.9)	Ref
≤ 500	477 (48.2)	312 (50.6)	200 (54.1)	1.1 (0.9–1.5)
Received social support in the past 12 months				
Yes	956 (95.2)	599 (96.1)	354 (93.7)	Ref
No	48 (4.8)	24 (3.9)	24 (6.3)	1.7 (1.1–3.0)

*Abbreviations*: *CI*, *confidence interval; GHQ*, *general health questionnaire; OR*, *odds ratio; PLHIV*, *people living with HIV*.

^*^ *Chi-square test was used for the comparisons*.

### Experiences of stigma and discrimination

**Table 2** shows the prevalence rates of AIDS-related stigma and discrimination experienced in different settings including home, community, workplace, religious settings, and healthcare facilities in the past 12 months. The common stigma and discrimination experiences included being gossiped about (42.3%), being verbally harassed and/or threatened (20.7%), being refused employment or job description/the nature of their work has been changed (13.2%), being subjected to psychological pressure by their spouse (12.1%), and having lost a job or another source of income (12.1%) as a result of their HIV positive status.

**Table 2:** Comparisons of stigma and discrimination experienced in communities in the past 12 months among PLHIV with lower and higher levels of mental disorders.

*Stigma and discrimination experiences*		Total score of GHQ-12		
	Total	≤ 3	≥ 4	
	*n* (%)	*n* (%)	*n* (%)	OR (95% CI)
Excluded from social gatherings or activities				
No	936 (93.1)	592 (94.9)	341 (90.2)	Ref
Yes	69 (6.9)	32 (5.1)	37 (9.8)	2.0 (1.2–3.3)
Excluded from religious activities				
No	982 (97.7)	613 (98.2)	366 (96.8)	Ref
Yes	23 (2.3)	11 (1.8)	12 (3.2)	1.8 (0.8–4.2)
Excluded from family activities (e.g. cooking, eating, sleeping together, etc.)				
No	957 (95.2)	601 (96.3)	353 (93.4)	Ref
Yes	48 (4.8)	23 (3.7)	25 (6.6)	1.9 (1.1–3.3)
Aware of being gossiped about				
No	580 (57.7)	396 (63.5)	183 (48.4)	Ref
Yes	425 (42.3)	228 (36.5)	195 (51.6)	1.9 (1.4–2.4)
Verbally insulted, harassed and/or threatened				
No	797 (79.3)	520 (83.3)	274 (72.5)	Ref
Yes	208 (20.7)	104 (16.7)	104 (27.5)	1.9 (1.4–2.6)
Physically harassed and/or threatened				
No	925 (92.0)	595 (95.4)	327 (86.5)	Ref
Yes	80 (8.0)	29 (4.6)	51 (13.5)	3.2 (2.0–5.1)
Physically assaulted				
No	984 (97.9)	615 (98.6)	366 (96.8)	Ref
Yes	21 (2.1)	9 (1.4)	12 (3.2)	2.2 (0.9–5.4)
Subjected to psychological pressure by your husband/wife or partner				
No	883 (87.9)	578 (92.6)	302 (79.9)	Ref
Yes	122 (12.1)	46 (7.4)	76 (20.1)	3.2 (2.1–4.7)
Experienced sexual rejection as a result of HIV positive status				
No	931 (92.6)	593 (95.0)	335 (88.6)	Ref
Yes	74 (7.4)	31 (5.0)	43 (11.4)	2.5 (4.5–4.0)
Discriminated against by other PLHIV				
No	922 (91.8)	576 (92.3)	343 (91.0)	Ref
Yes	82 (8.2)	48 (7.7)	34 (9.0)	1.2 (0.8–1.9)
Members of household experienced discrimination as a result of HIV status				
No	909 (90.6)	575 (92.4)	331 (87.6)	Ref
Yes	94 (9.4)	47 (7.6)	47 (12.4)	1.7 (1.1–2.7)
Forced to change your place of residence or unable to rent accommodation				
No	973 (96.8)	610 (97.8)	360 (95.2)	Ref
Yes	32 (3.2)	14 (2.2)	18 (4.8)	2.2 (1.1–4.4)
Lost a job or another source of income because of HIV status				
No	873 (87.0)	560 (89.7)	310 (82.2)	Ref
Yes	131 (13.0)	64 (10.3)	67 (17.8)	1.9 (1.3–2.7)
Refused employment or a work opportunity because of HIV status				
No	872 (86.8)	569 (91.2)	300 (79.4)	Ref
Yes	133 (13.2)	55 (8.8)	78 (20.6)	2.7 (1.9–3.9)
Nature of work been changed, or been refused promotion as a result of HIV status				
No	872 (86.8)	564 (90.4)	305 (80.7)	Ref
Yes	133 (13.2)	60 (9.6)	73 (19.3)	2.3 (1.6–3.3)
Dismissed or prevented from attending an educational institution because of HIV				
No	891 (88.7)	576 (92.3)	312 (82.5)	Ref
Yes	114 (11.3)	48 (7.7)	66 (17.5)	2.5 (1.7–3.8)
Your child had been prevented from attending school because of your HIV status				
No	954 (95.0)	603 (96.6)	348 (92.3)	Ref
Yes	50 (5.0)	21 (3.4)	29 (7.7)	2.4 (1.3–4.3)
Denied health services because of HIV status				
No	996 (99.2)	622 (99.7)	371 (98.4)	Ref
Yes	8 (0.8)	2 (0.3)	6 (1.6)	5.0 (1.1–25.0)

*Abbreviations*: *CI*, *confidence interval; GHQ*, *general health questionnaire; OR*, *odds ratio; PLHIV*, *people living with HIV*.

^*^ *Chi-square test or Fisher’s Exact test was used as appropriate for the comparisons*.

**Table 2** also shows that most of the AIDS-related stigma and discrimination experiences were significantly associated higher levels of mental disorders (GHQ-12≥ 4). For examples, PLHIV with a total GHQ-12 score of ≥ 4 were significantly more likely to report being physically harassed and/or threatened (OR = 3.2, 95% CI = 2.0–5.1), being subjected to psychological pressure by their spouse (OR = 3.2, 95% CI = 2.1–4.7), being forced to change place of resident or unable to rent accommodation (OR = 2.2, 95% CI = 1.1–4.4), being refused employment (OR = 2.7, 95% CI = 1.9–3.9), having nature of work changed or being refused promotion (OR = 2.3, 95% CI = 1.6–3.3), or being prevented from attending an educational institution (OR = 2.5, 95% CI = 1.7–3.8) in the past 12 months as a result of their HIV positive status.

### Internal stigma

Internal stigma experienced in the past 12 months and its bivariate relationship with levels of mental disorders are shown in **Table 3**. The most common internal stigma included feeling guilty (53.1%), feeling self-blamed (46.3%), feeling ashamed (45.9%), and feeling that they should be punished (25.6%). Most of the internal stigma variables were positively associated with levels of mental disorders (GHQ-12 ≥4). For examples, PLHIV with a total GHQ-12 score of ≥4 were significantly more likely to report feeling ashamed (OR = 2.1, 95% CI = 1.6–2.8), having low self-esteem (OR = 2.4, 95% CI = 1.8–3.2), or feeling suicidal (OR = 4.5, 95% CI = 2.4 = 8.4) compared to those with a total GHQ-12 score of ≤3. They were also significantly more likely to report isolating themselves from family and/or friends (OR = 3.2, 95% CI = 1.7–5.8), or making a decision to stop working (OR = 3.2, 95% CI = 1.7–5.8).

**Table 3:** Comparisons of internal stigma experienced in the past 12 months among PLHIV with lower and higher levels of mental disorders.

*Internal stigma*		Total score of GHQ-12		
	Total	≤ 3	≥ 4	
	*n* (%)	*n* (%)	*n* (%)	OR (95% CI)
I feel ashamed				
No	543 (54.1)	381 (61.1)	160 (42.4)	Ref
Yes	461 (45.9)	243 (38.9)	217 (57.6)	2.1 (1.6–2.8)
I feel guilty				
No	471 (46.9)	326 (52.2)	143 (37.9)	Ref
Yes	533 (53.1)	298 (47.8)	234 (62.1)	1.8 (1.4–2.3)
I blame myself				
No	539 (53.7)	370 (59.3)	168 (44.6)	Ref
Yes	465 (46.3)	254 (40.7)	209 (55.4)	1.8 (1.4–2.3)
I blame others				
No	834 (83.2)	531 (85.2)	300 (79.6)	Ref
Yes	169 (16.8)	92 (14.8)	77 (20.4)	1.5 (1.1–2.1)
I have low self-esteem				
No	779 (77.7)	520 (83.5)	256 (67.9)	Ref
Yes	224 (22.3)	103 (16.5)	121 (32.1)	2.4 (1.8–3.2)
I feel I should be punished				
No	691 (68.8)	464 (74.4)	225 (59.7)	Ref
Yes	313 (31.1)	160 (25.6)	152 (40.3)	2.0 (1.5–2.6)
I feel suicidal				
No	955 (95.1)	610 (97.8)	342 (90.7)	Ref
Yes	49 (4.9)	14 (2.2)	35 (9.3)	4.5 (2.4–8.4)
I chose not to attend social gathering				
No	903 (89.9)	566 (90.7)	335 (88.6)	Ref
Yes	102 (10.1)	58 (9.3)	43 (11.4)	1.3 (0.8–1.9)
I isolated myself from my family and/or friends				
No	936 (93.1)	599 (96.0)	334 (88.4)	Ref
Yes	69 (6.9)	25 (4.0)	44 (11.6)	3.2 (1.9–5.3)
I made the decision to stop working				
No	956 (95.1)	607 (97.3)	347 (91.8)	Ref
Yes	49 (4.9)	17 (2.7)	31 (8.2)	3.2 (1.7–5.8)
I decided not to apply for a job or a promotion				
No	944 (93.9)	588 (94.2)	354 (93.7)	Ref
Yes	61 (6.1)	36 (5.8)	24 (6.3)	1.1 (0.7–1.9)
I withdrew from education/training opportunity				
No	959 (95.6)	593 (95.2)	364 (96.6)	Ref
Yes	44 (4.4)	30 (4.8)	13 (3.4)	1.4 (0.7–2.8)
I decided not to get married				
No	573 (57.1)	339 (54.5)	232 (61.4)	Ref
Yes	430 (42.9)	283 (45.5)	146 (38.6)	1.3 (1.1–1.7)
I decided not to have sex				
No	648 (64.5)	403 (64.6)	243 (64.3)	Ref
Yes	357 (35.5)	221 (35.4)	135 (35.7)	1.0 (0.8–1.3)
I decided not to have (more) children				
No	406 (40.4)	263 (42.1)	142 (37.6)	Ref
Yes	599 (59.6)	361 (57.9)	236 (62.4)	1.2 (0.9–1.6)
I avoided going to a local clinic and/or hospital				
No	977 (97.2)	608 (97.4)	366 (96.8)	Ref
Yes	28 (2.8)	16 (2.6)	12 (3.2)	1.2 (0.6–2.7)

*Abbreviations*: *CI*, *confidence interval; GHQ*, *general health questionnaire; OR*, *odds ratio; PLHIV*, *people living with HIV*.

^*^ *Chi-square test or Fisher’s Exact test was used as appropriate for the comparisons*.

### Fear of stigma and discrimination

**Table 4** presents the prevalence rates of fear of AIDS-related stigma and discrimination and their association with levels of mental disorders. PLHIV in this study experienced several forms of fear including fear of being gossiped about (34.5%), fear of being rejected for sexual intimacy (16.2%), fear of being verbally insulted (15.5%), and fear of being physically assaulted (13.9%). PLHIV with higher levels of mental disorders (GHQ-12≥ 4) were significantly more likely to report having been fearful of being gossiped about (OR = 2.2, 95% CI = 1.7–29), being verbally insulted (OR = 2.5, 1.9–3.3), being physically harassed (OR = 2.6, 95% CI = 2.6, 95% CI = 1.8–3.6), and being physically assaulted (OR = 2.6, 95% CI = 1.8–3.8).

**Table 4:** Comparisons of fear of stigma and discrimination experienced in the past 12 months among PLHIV lower and higher levels of mental disorders.

*Fears of stigma and discrimination*		Total score of GHQ-12		
	Total	≤ 3	≥ 4	
	*n* (%)	*n* (%)	*n* (%)	OR (95% CI)
*Having been fearful of the following things happening to you*:				
Being gossiped about				
No	658 (65.5)	451 (72.3)	204 (54.0)	Ref
Yes	347 (34.5)	173 (27.7)	174 (46.0)	2.2 (1.7–2.9)
Being verbally insulted, harassed, or threatened				
No	758 (75.4)	511 (81.9)	244 (64.6)	Ref
Yes	247 (24.6)	113 (18.1)	134 (35.4)	2.5 (1.9–3.3)
Being physically harassed and/or threatened				
No	849 (84.5)	557 (89.3)	289 (76.5)	Ref
Yes	156 (15.5)	67 (10.7)	89 (23.5)	2.6 (1.8–3.6)
Being physically assaulted				
No	865 (86.1)	565 (90.5)	297 (78.6)	Ref
Yes	140 (13.9)	59 (9.5)	81 (21.4)	2.6 (1.8–3.8)
Being rejected to have sexually intimate with				
No	837 (83.8)	527 (84.7)	307 (82.1)	Ref
Yes	162 (16.2)	95 (15.3)	67 (17.9)	1.2 (0.9–1.7)

*Abbreviations*: *CI*, *confidence interval; GHQ*, *general health questionnaire; OR*, *odds ratio; PLHIV*, *people living with HIV*.

^*^ *Chi-square test was used for the comparisons*.

### Results of bivariate and multivariate logistic regression analyses

As shown in **Table 5**, in bivariate logistic regression analyses, PLHIV with higher levels of mental disorders (GHQ-12≥ 4) were significantly more likely to have higher levels of all forms of AIDS-related stigma and discrimination with all *p*-values< 0.001. After controlling for other co-variates in the multivariate logistic regression models, PLHIV with a total GHQ-12 score of ≥ 4 remained significantly more likely to have experienced higher levels of AIDS-related stigma and discrimination (AOR = 1.9, 95% CI = 1.4–2.6), higher levels of internal stigma (AOR = 1.7, 95% CI = 1.2–2.3), and higher levels of fear of AIDS-related stigma and discrimination (AOR = 1.5, 95% CI = 1.1–2.2) in the past 12 months.

**Table 5:** Results of bivariate and multivariate logistic regression analyses of the association between stigma and discrimination with levels of mental disorders among PLHIV.

*Stigma and discrimination scales*	Total score of GHQ-12			
	≤ 3	≥ 4		
	*n* (%)	*n* (%)	OR (95% CI)	AOR (95% CI)^†^
Total score of stigma and discrimination experience				
≤ 2	468 (75.2)	210 (56.1)	Ref	Ref
≥ 3	154 (24.8)	164 (43.9)	2.4 (1.8–3.1)***	1.9 (1.4–2.6)***
Total score of internal stigma				
≤ 4	434 (70.1)	196 (52.1)	Ref	Ref
≥ 5	185 (29.9)	180 (47.9)	2.2 (1.7–2.8) ***	1.7 (1.2–2.3) **
Total score of fear of stigma and discrimination				
≤ 1	492 (79.1)	237 (63.4)	Ref	Ref
≥ 2	130 (20.9)	137 (36.6)	2.2 (1.6–2.9) ***	1.5 (1.1–2.2)*

*Abbreviations*: *AOR*, *adjusted odds ratio; CI*, *confidence interval; GHQ*, *general health questionnaire; OR*, *odds ratio; PLHIV*, *people living with HIV*.

^†^ *Socio-demographic variables that were associated with GHQ-12 in bivariate analyses at a level of p≤ 0*.*10 (gender*, *education level*, *marital status*, *employment status*, *income*, *and spouse’s HIV sero-status) were included in the multivariate logistic regression models*.

^*^ *p< 0*.*05;*

^**^ *p< 0*.*01;*

^***^ *p< 0*.*001*.

## Discussion

This study examined the prevalence rates of different forms of AIDS-related stigma and discrimination and their relationship with mental disorders among PLHIV in Cambodia. Consistent with previous studies, this study demonstrates that PLHIV continue to experience significant levels of various forms of stigma and discrimination in familial and community interactions that impact a broad range of aspects in their daily lives [52–54], although they manifest differently and in varying degrees in different settings and countries [55].

The People Living with HIV Stigma Index has been used to measure AIDS-related stigma and discrimination in different countries and regions enabling international comparisons of the prevalence rates of stigma and discrimination across the world. Using this stigma index, the Asia-Pacific regional analysis found that Cambodia is among the countries in the region with relatively lower proportions of PLHIV who reported experiencing AIDS-related stigma and discrimination than many other countries in the region, and the proportions were similar to findings in this study [55]. For examples, reports of experiences of physical harassment and threats ranged from 4% in Cambodia to 22% in Pakistan; this prevalence was 8% among Cambodian PLHIV in this study. The proportion of PLHIV who reported having been forced to move or had been unable to rent accommodation during the past 12 months as a result of their HIV-positive status ranged from 5% in Sri Lanka to 20% in Pakistan; in the regional study, this prevalence was 12% among Cambodian PLHIV compared to only 3% among Cambodian PLHIV in this study.

The prevalence of reported internal stigma among PLHIV in this study was common but relatively lower than the rates documented in other countries in the region, ranging from 3% to 60%. The Asia-Pacific regional analysis of People Living with HIV Stigma Index [55] demonstrated that the proportion of PLHIV who reported feeling ashamed ranged from 54% in Sri Lanka to 76% in Pakistan. This prevalence was 62% among Cambodian PLHIV in the regional report compared to 46% in this study. The proportion of PLHIV who reported feeling self-blamed was also high, ranging from 51% in Sri Lanka to 80% in Fiji. In the Asia-Pacific regional study, 16% of PLHIV in Cambodia reported feeling suicidal in the past 12 months compared to 48% in China. The most common internal stigma found in the Asia-Pacific regional study was feeling of low self-esteem which ranged from 22% in Sri Lanka to 81% in Myanmar compared to 45% in Cambodia.

Furthermore, remarkably lower proportions of PLHIV in this study reported experiences of several forms of AIDS-related stigma and discrimination in the past 12 months compared to findings from a recent study in South Africa [52]. For examples, the proportion of PLHIV who reported being excluded from social gathering or activities was 7% among Cambodian PLHIV in this study compared to 19% among PLHIV in South Africa. Similarly, the proportion of PLHIV who reported being verbally insulted, harassed, or threatened was 21% among Cambodian PLHIV in this study compared to 28% among PLHIV in South Africa. Regarding internal stigma, only 5% of Cambodian PLHIV in this study reported feeling suicidal in the past 12 months compared to 15% among PLHIV in South Africa. Moreover, only 22% of Cambodian PLHIV in this study reported having low self-esteem compared to 32% among PLHIV in South Africa [52].

Although PLHIV in Cambodia are likely to experience lower levels of AIDS-related stigma and discrimination than those living in other countries, these experiences seem to have significant impacts on their mental health status. This finding is in line with findings from several studies in different settings and populations. AIDS-related stigma and discrimination were found to be associated with major depressive disorders in studies in Uganda [15,17] and South Africa [40]. In a study among Black men living with HIV in the United States, several types of perceived AIDS-related discrimination was found to contribute to poor mental health including depression and posttraumatic stress disorder [41]. The relationship between AIDS-related stigma and discrimination and mental health problems has also been reported in several studies in China [33,34,56], India [39], Thailand [57], and many countries across Africa, Asia-Pacific, Europe, Latin America, and North America [5].

AIDS-related stigma and discrimination may be one explanation for the disparities in mental health conditions of PLHIV and general population. The relationship of levels of AIDS-related stigma and discrimination with mental disorders in this study is consistent with bio-psychosocial models that conceptualize stigma and discrimination as a stressor [58,59]. A meta-analysis also concluded that individuals who experience chronic stigma and discrimination are vulnerable to poor mental health including anxiety, depression, and distress [60]. Chronic discrimination creates a hostile living environment that can lead to wear and tear of protective mechanisms and over time, a lower capacity for coping with new stressors [41]. A possible explanation for the relationship can also be drawn from socio-psychological studies on theories of minority stress [61]. PLHIV may experience excess stress from stigma and discrimination, which creates a hostile living environment and in turn, lead to mental disorders [41].

Findings from this study should be interpreted in light of some methodological limitations. The first limitation concerns the representativeness of the study population. The study samples were recruited from provinces where the SAHACOM, a comprehensive community-based project aiming to improve overall health and quality of life of PLHIV, has been implemented. The levels of AIDS-related stigma and discrimination as well as mental wellbeing reported in this study may therefore represent a more optimistic view than in other areas of Cambodia. Secondly, our measure of mental disorders using GHQ-12 was not a diagnostic instrument and can only be interpreted as symptoms of negative affects or emotional distress not as specific mental illnesses per se. Thirdly, the cross-sectional design precludes causal interpretation of the findings. Longitudinal studies are needed to examine the causal relationship of the existence of AIDS-related stigma and discrimination with mental health of PLHIV. Finally, findings from this study may be limited by the questionnaire survey measures that can lead to social desirability biases.

Despite these limitations, we believe that findings from this study carry important implications for health policies and intervention services for improving health and quality of life of PLHIV in Cambodia. The significant relationship of AIDS-related stigma and discrimination with mental disorders indicates a need for community-based interventions to reduce stigma and discrimination in the general public and to help PLHIV to cope with these stressful situations. A review of strategies for stigma reduction suggest promising results of legal protection, provision of ART, and introduction of quality HIV care in reducing public fear of HIV [62].
